# Spontaneous associative thought may facilitate scene-gist memory via implicit scene-labeling

**DOI:** 10.3758/s13421-024-01672-y

**Published:** 2024-12-02

**Authors:** Shira Baror, Elissa Aminoff, Yoed N. Kenett

**Affiliations:** 1 The Edmond and Lily Safra Center for Brain Sciences, The Suzanne and Charles Goodman Brain Sciences Building, Edmond J. Safra Campus, The Hebrew University, 9190401 Jerusalem, Israel; 2 Department of Psychology, Fordham University, Bronx, NY USA; 3 Faculty of Data and Decision Sciences, Technion — Israel Institute of Technology, Haifa, Israel

**Keywords:** Associative thought, Creativity, Scene perception, Scene memory

## Abstract

Spontaneous associative processes (e.g., mind wandering, spontaneous memory recollection) are prevalent in everyday life, yet their influence on perceptual scene memory is under debate. Given that scene perception involves extraction of contextual associations, we hypothesized that associative thought would enhance scene memory by promoting encoding of contextual associations. In an online experiment (N = 75), participants viewed scenes, and following each scene either generated chained-free associations (associative processing), or, as control, listed words that begin with a specific letter (phonological processing). Scene memory was tested after an intermediate creativity task, which is also shown to rely on associative processes. Results revealed that associative thought, regardless of its conceptual (semantic) distances between responses, enhanced scene-gist memory, but hampered memory of scene details, implying that associative thought facilitates contextual encoding. In a follow-up experiment (N = 74), we found that the effect of associative thought on scene-gist memory was mediated by scene labeling. When participants were asked to explicitly label the scene before completing an associative processing or a phonological processing task, scene-gist memory was prioritized at the expense of scene details, eliminating the memory differences between tasks. These findings imply that labeling past perceived scenes, whether explicitly or implicitly during associative thought, facilitates scene-gist memory. Lastly, in both experiments, creativity was not correlated with scene memory but was positively correlated with the semantic distances between scene-based associations, extending past findings that link creativity with the breadth of associative processes. Together, these findings highlight the likely effect of post-perceptual associative processes on higher-order cognitive functions, such as memory consolidation and creative thought.

## Introduction

Imagine a scenario of driving home from the beach after enjoying a beautiful sunset. While driving home you think of the sunset, of how you wish your ex-partner was there with you, which then triggers the thought of your ex-partner’s dog, it’s loud barking at birds, and how do birds fly anyway. Now imagine an alternative scenario, of leaving the beach, entering the car, and being caught up in a long work-related conversation. How likely are you to remember the beautiful sunset in each of these scenarios? The scenes we engage in spontaneously trigger associated thoughts and memories (Baror & He, 2021; Gilmore et al., 2016). These associative processes are elicited unintentionally, taking the form of spontaneous undirected flow of thoughts (Baror et al., 2021), often connecting the current with past experiences and simulations of the future (Christoff et al., 2016). Yet it is an open question how these thoughts influence subsequent memory. The current research aimed to test whether associative thought that emerges following scene perception enhances scene memory.

On the one hand, associative thought following scene perception can compromise scene memory. Engaging in associative thought that is semantically related to the previously presented scene has been shown to interfere with memory of scene details (Melcher & Murphy, 2011). Spontaneous associative thought has also been linked with reduced memory (Blonde et al., 2022), though this negative effect is attenuated when thoughts are stimulus dependent. Furthermore, one’s mental context drifts in time (DuBrow et al., 2017), sometimes to completely unrelated topics, and a dramatic change in the semantic content from the just-observed scene (e.g., answering an urgent work-related phone call shortly after viewing the sunset) may reduce the ability to rely on that content as a contextual pillar at retrieval.

On the other hand, spontaneous associative thought may facilitate scene memory. Support for this prediction comes from a recent study (Baror et al., 2022) which showed that, over time, engaging in an associative mindset while viewing scenes enhances scene perception. This work further suggested that scene perception and associative thought share an associative foundation. This has been pointed out in the past in a review suggesting that scene perception and spontaneous associative thought converge on activity in shared brain regions within the Default Mode Network (DMN), which are also activated by associative processing (Baror et al., 2021). Scene perception relies on associating individual elements (Kim & Biederman, 2011), learning that the appearance of one predicts the appearance of the other. The mere acquiring of such associative information between features, even if they are semantically meaningless, is found to engage scene-selective brain regions (Aminoff & Tarr, 2015; Aminoff et al., 2007). These findings imply that the identification of associative links between separate elements is what inherently drives scene perception.

Importantly, scene perception involves not only the links between individual elements, but of extracting the global gist as well (Bar, 2004; Torralba et al., 2006). It is found that long-term contextual associations, (e.g., expecting a cow or a chicken to be found in the farm) modulate activity in scene-selective brain regions (Bar & Aminoff, 2003; Bar et al., 2007). Complementarily, associations that are spontaneously evoked during memory encoding (Peters et al., 2009) or spontaneous episodic thought that relates to one’s own past experiences (Gilmore et al., 2016; Szpunar et al., 2009) activate scene-selective brain regions, lending support to the idea that associative thought is tightly linked with scene construction, potentially by promoting the consolidation and representation of contextual information. This is in line with recent frameworks postulating that spontaneous associative thought serves as a spontaneous memory replay of past events (Mildner & Tamir, 2019; Mills et al., 2018). Therefore, our primary hypothesis is that associative thought that occurs following scene perception, as in the beach scenario described above, can potentially improve memory of scenes.

Beyond its relation to how we make sense of scenes, associative processes have been robustly linked with creative abilities and creative behaviors. Numerous creativity studies emphasize that associativity is at the core of creativity (Beaty & Kenett, 2023; Benedek et al., 2023; Marron et al., 2018; Mednick, 1962; Merseal et al., 2023; Raffaelli et al., 2023), because creativity requires associating previously unrelated or distant concepts for the generation of a new idea. These studies link creativity with the ability to travel larger conceptual distances in one’s memory when engaging in associative tasks (Gray et al., 2019). In a recent review, Beaty and Kenett (2023) highlight the role of associative thought as a core mechanism of creative thinking, and illustrate how computational methods have paved the way to study associative thought in creativity. Specifically, a new measure called “Forward Flow” (FF; Gray et al., 2019) has been recently in use to quantify the conceptual distance between associated concepts. This measure is primarily used when participants are given a concept word and are asked to provide an association with that concept, an association with the first association, and so forth, such that a chain of free associations in created (Beaty et al., 2021; Gray et al., 2019). This method empirically quantifies one’s ability to search one’s memory and relates such memory search to individual differences in creativity (Merseal et al., 2023), with higher FF scores indexing broader associative thought (see also Olson et al., 2021). Across multiple samples, the FF score has been related to individual differences in divergent thinking, a core aspect of creative thinking which pertains to one’s ability to come up with as many unique answers to a given question (Acar & Runco, 2019; Runco & Jaeger, 2012). In a recent study, Beaty et al. (2021) have shown that FF is a strong independent predictor of divergent thinking, alongside intelligence, further highlighting the significance of associative thought in creativity.

In addition to our primary hypothesis, here we leveraged the established link between associative thought and creativity to test two secondary hypotheses: First, that higher creativity will be linked with more distant associations one provides when perceiving a scene. Second, that higher creativity will be linked with better scene memory via enhanced associative thought. This hypothesis stems from the fact that both scene perception and creativity separately rely on associative processes, which raises the intriguing possibility that associations mediate a link between creativity and scene memory.

## Experiment 1

### Methods

#### General paradigm

**Fig. 1:**
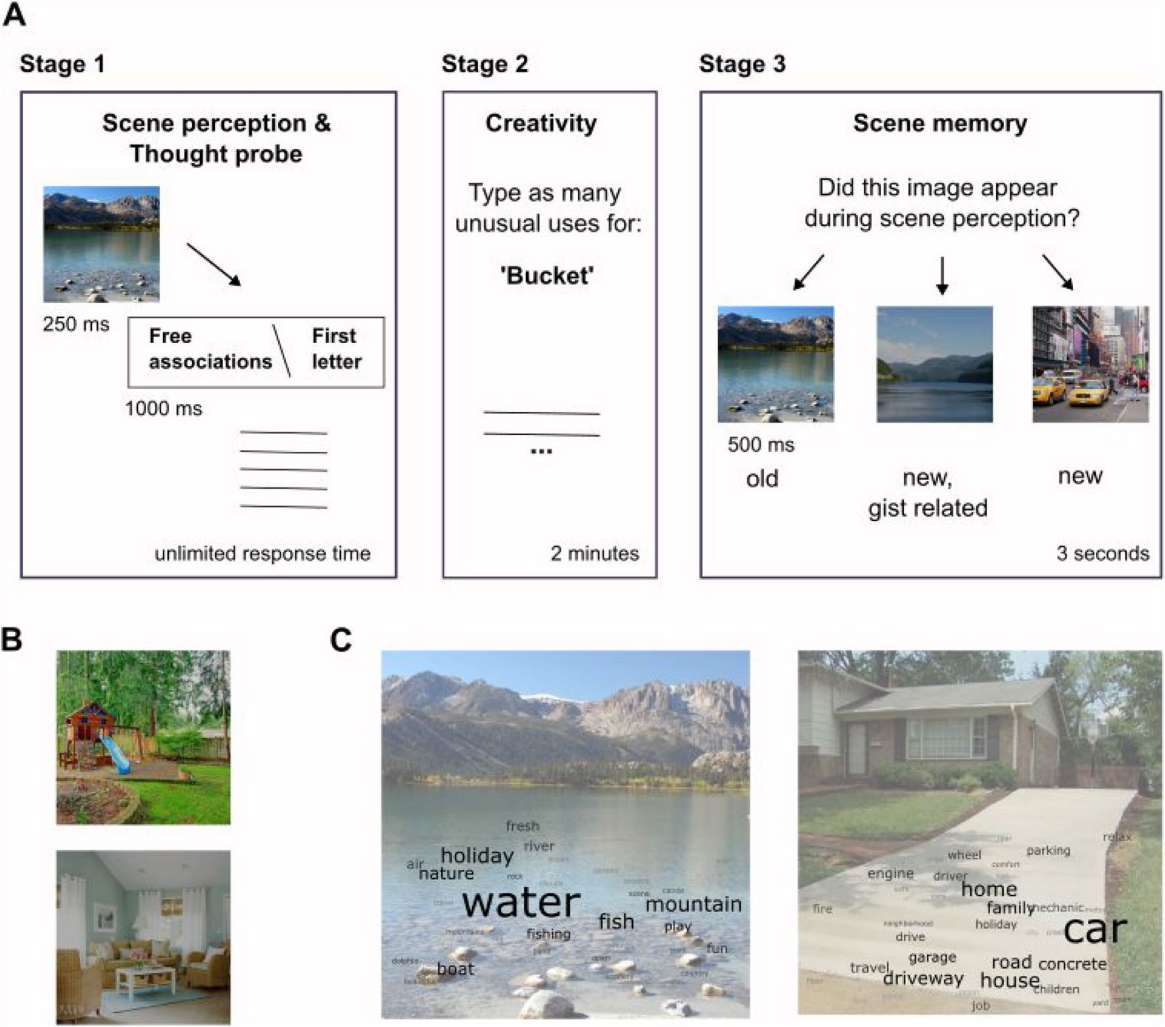
Paradigm and chained-free associations. **(A)** Paradigm. The experiment included three stages: Scene perception and thought probe (either “free associations,” indicating the associative thought task condition, or “first letter,” indicating the phonological task condition. Twenty-eight trials were presented in each cue condition (with unlimited response time), a creativity-related alternative uses task (three trials, each limited to 2 min response time), and a final scene memory stage. Fifty percent of the trials in the memory stage presented scenes that appeared during the scene perception and thought probe stage, with equal representation of scenes that were followed by the free associations cue and scenes that were followed by the first letter cue. Twenty-five percent of the scenes were new, yet gist related to the scenes that appeared during the scene-perception stage, with an equal proportion of scenes related to either cue conditions. Finally, 25% of trials were completely new scenes. **(B)** Outdoor and indoor scene examples. **(C)** Chained free associations examples. Two scene examples are depicted, over which are superimposed the frequent associations provided following viewing each scene. Letter size represents frequency across participants. Word-clouds are generated via freewordcloudgenerator.com

A three-stage experiment was designed (Fig. 1). In the first stage, participants viewed scenes, and after each scene performed either an associative thought task, which engaged them in associative processing, or a phonological fluency task, which served here as a control. In the second stage, participants engaged in the alternative uses task (Guilford, 1967), which measures divergent thinking abilities. In the last stage, a surprise scene-memory task was introduced, in which participants were asked to determine whether presented scenes appeared during the first stage of the experiment or not. Our primary prediction was that scene-memory will be better for scenes that were followed by associative thought compared with scenes that were followed by the control task. This second stage primarily functioned as a buffer between the first and third stages, but also served to examine our secondary predictions: (1) that the semantic distance between associations one generates during associative thought will correlate with their creative abilities, and (2) that participants who show high creative abilities will show superior scene memory.

#### Participants

One hundred participants (31 female) from the UK and USA were recruited via Prolific. Sample size was computed based on power analysis planned to obtain a medium to large effect sizes of 0.4 in paired t-tests, with power of 0.95. This was based on large effects found in our previous work (Baror et al., 2022). We increased the sample size suggested by the power analysis from 59 to 100, given the additional planned correlation analysis that looks at individual differences beyond differences between the groups. This is in line with an additional power analysis planned to obtain a medium to large correlation value of 0.4 in bivariate correlation analysis, with power of 0.95.

Eligibility criteria limited participation only to people who were native English speakers, and did not have neurological deficits, learning disabilities, attention disorders, or compromised mental health conditions. We also limited participation to desktop computers only. Mean age was 39 years (SD = 13.27 years). Eleven participants did not complete the experiment and an additional 14 participants completed the experiment but did not follow instructions properly (i.e., did not consistently respond according to the task instructions and cues in the first stage of the experiment). Those participants were excluded from all analyses. Thus, the final sample size of the experiment was 75 participants (mean age = 37 years, SD = 11.5 years, 22 female). All parts of the experiment were programmed and administered via Psychopy (Peirce et al., 2019), and the experiment was hosted online via Pavlovia (https://pavlovia.org). The experiment extended ~ 40 min and participants were paid 4 pounds for their time. The experiment was approved by the Institutional Review Board of Technion, Israel Institute of Technology.

#### Stimuli

Scene stimuli were taken from the “BOLD5000” dataset (Chang et al., 2019). This dataset includes high-resolution, color photographs depicting common everyday scenes (e.g., street, kitchen, etc.), all originally labeled in the “SuN” database (Xiao et al., 2010). For the purpose of the current study, the stimuli sample included pairs of scene images that share the same gist (e.g., two different scene images of a beach), and that do not overlap with other pairs (e.g., only two beaches in the entire sample). An equal proportion of indoor and outdoor naturalistic scenes were included. The stimuli sample ultimately included a total of 70 pairs. Scenes’ size was 375 × 375 pixels.

#### Procedure

Participants began the experiment by signing an online consent form and reading the instructions, which informed them that the experiment will involve three tasks, and that each task will be explained at its turn.

##### Stage 1: Scene perception and thought probe task

In this task, participants were presented with an image of a scene and were required to either generate chained-free associates or perform a phonological fluency task. Each scene was presented for 250 ms, after which a cue appeared on-screen for 1 s, indicating which of two possible tasks should be performed. Cues were presented after the scene disappeared from the screen, such that during scene viewing participants did not know which task they were about to perform. Cue condition was randomly interleaved. The time to provide five responses on each trial was not limited and was measured in the analysis as a proxy for task difficulty. A total of 56 scene images were presented in this stage, 28 images in each task condition.

##### Associative thought task

If a “Free Associations” cue appeared, participants were asked to perform the chained-free associations task, in which they had to generate an association with the scene. After that association was provided, participants then had to generate an association with the word that they just typed in. This repeated process created a chain of five associations, starting from the scene. For example: After seeing a scene depicting a ski site, participants could have responded with: “snow- ice- temperature- fire- marshmallow.” They were instructed to only type single words, and not to type proper nouns (such as names, brands, etc.), which are not commonly included in text corpora, used to compute FF.

##### Phonological fluency task

If a ‘First Letter’ cue appeared, participants were asked to perform the phonological fluency task in which they were asked to provide five words that begin with a specific letter, irrespective of the scene that was presented. Participants were instructed that the words they generate in this task did not need to be associatively related to one another, and that they only needed to begin with the cued letter. For example, if the cue “First letter” appeared, and below it the letter “B” appeared, participants could have responded with: “balloon- bulldozer- bark- bowling- banana.”

##### *Stage 2: Creativity task*

In the second stage, the Alternative Uses Task (AUT) was implemented. In this task, the word of an object was presented and participants were instructed to generate as many possible unusual uses to that object, in line with previous protocols (Runco, 2004). For example, for the word “sock,” participants could suggest the use of a hand puppet, and for the word “bucket,” participants could suggest a helmet. Participants were specifically instructed to come up with creative uses, which were defined in the study as uses that strike people as clever, unusual, interesting, uncommon, humorous, innovative, or different. Participants provided their responses by typing them via the keyboard. A total of three trials were included in the task, depicting the words Sock, Bucket, and Candle, randomly ordered across participants. For each word, participants were given 2 min to provide as many alternative uses as possible. After 6 min this experimental stage concluded.

##### *Stage 3: Scene memory task*

The experiment concluded with a scene memory test, testing participants’ memory of the images that appeared during the first experimental stage. Scene images were consecutively presented on-screen for 500 ms, and participants were asked to make a two-alternative forced choice and decide whether the presented scene appeared during the first stage of the experiment or not. Half the trials in this stage depicted scenes that appeared during the first stage, and half were completely new, thus preventing response-related bias. Crucially, of the images that appeared during the first stage, half appeared prior to the associative thought task, and half appeared prior to the phonological fluency task. Of the new images, half were completely new images and half were images that shared the same gist as images that appeared during the first stage, with an equal proportion of gist relatedness to scenes preceding the associative thought task and gist relatedness to scenes preceding the phonological fluency task. A total of 56 scene images were presented at this stage.

### Data analysis

#### Stage 1

Results of stage 1 were analyzed separately for the associative thought task and the phonological fluency task. In each task, we calculated the mean reaction time (RT) it took the participant to provide five responses.

##### Forward Flow analysis

To quantify the semantic distance in associative thought, we leveraged the recently developed “Forward Flow” metric (FF; Gray et al., 2019), which uses latent semantic analysis to evaluate the semantic evolution of thoughts over time, and adapted it to scene-based associative thinking. This task provides a quantitative way to empirically measure how one searches through one’s memory. FF is calculated as the semantic distance, or dissimilarity, between concepts based on a semantic space derived from large textual corpora. To compute the semantic distance between the first associate and the scene, we used the pre-determined word label of the images given to them in the original “SuN” database (Xiao et al., 2010). We then averaged the pairwise FF score to compute an averaged FF score per scene and averaged this score over scenes to compute a participant-specific scene-perception FF score. To calculate the FF score, we used multiple semantic spaces and combined them into an average latent factor score (Beaty et al., 2021). A higher FF score indicates greater semantic distance between associations.

##### Forward Flow (FF) in the phonological fluency analysis

This is a control task as it involves verbal retrieval yet is not focused on semantic information. Although not focused on associative processing, the FF score was computed to these words as well. Considering that the answers provided in this task are not required to be semantically associated with one another, FF scores were expected to be larger in this task compared to the associative thought task, reflecting greater semantic distances between the words.

#### Stage 2

##### Creativity analysis

Performance in the alternative uses task was evaluated via an automated procedure using the AI-based scoring of divergent thinking (Grajzel et al., 2022). This automated analysis uses a pre-trained large language model (Organisciak et al., 2023), and is found to capture participants originality in the AUT (see also Beaty & Johnson, 2021; Dumas et al., 2021). It evaluates each participant’s response in the task such that higher scores indicate greater originality. Scoring individual responses was followed by averaging for each participant their creativity scores across all responses in all trials.

#### Stage 3

##### Scene memory analysis

Hit rate was computed for scenes that appeared in the scene-perception stage prior to each task condition. Correct rejection rate was computed for new scenes, while sub-grouping trials to three conditions: (i) completely new scenes, (ii) new scenes that were gist related (e.g., a different restaurant) to the scenes that appeared in the scene-perception stage prior to the free associations cue-condition, (iii) new scenes that were gist related to scenes that appeared in the scene-perception stage prior to the first-letter cue condition.

Additionally, signal detection parameters of sensitivity (D’) and criterion were computed, each corresponding to a different ability within the scene memory task. Sensitivity pertains to the ability to differentiate between old and new images and criterion pertains to the minimal internal threshold of certainty one relies on to decide that an image has appeared in the first part of the experiment. To compute theses parameters, we used the following formulas:

$${D}{^\prime}=zHits-zFalse\;Alarms$$

$$Criterion=-0.5*\left(zHits+zFalse\;Alarms\right)$$

Sensitivity and criterion were computed for each task condition, considering hit rate in old scenes and false alarm rate in new yet gist-related scenes. New scenes that were not gist related to scenes that appeared in the scene-perception stage were not included in the signal-detection analysis.

*Software information:* Computing the FF score was carried out using R scripts (software version 2024.04.0) provided via an open-access Open Science Framework (OSF) link by Gray et al. (2019). Analysis of memory performance as well as correlation between memory, creativity, and the FF scores were carried out using custom-made MATLAB scripts (software version 2024a), as well as using the SPSS software (software version 29.0.2.0 (20)).

### Results

We first evaluated whether the associative thought task and the phonological fluency task were of equivalent difficulty by comparing differences in the time it took to provide all five responses (i.e., RTs) between the tasks. A paired-samples *t*-test analysis revealed a null effect (associative thought task: mean RT = 33.17 s (SD = 14.74 s); phonological fluency task: mean RT = 36.52 s (SD = 42.45 s)), *t*_(74,1)_ = 1.16, *p* = 0.24, d = 0.13). Thus, there is no evidence that the tasks following scene perception differed in difficulty.

#### Associative thought and scene-gist memory

**Fig. 2:**
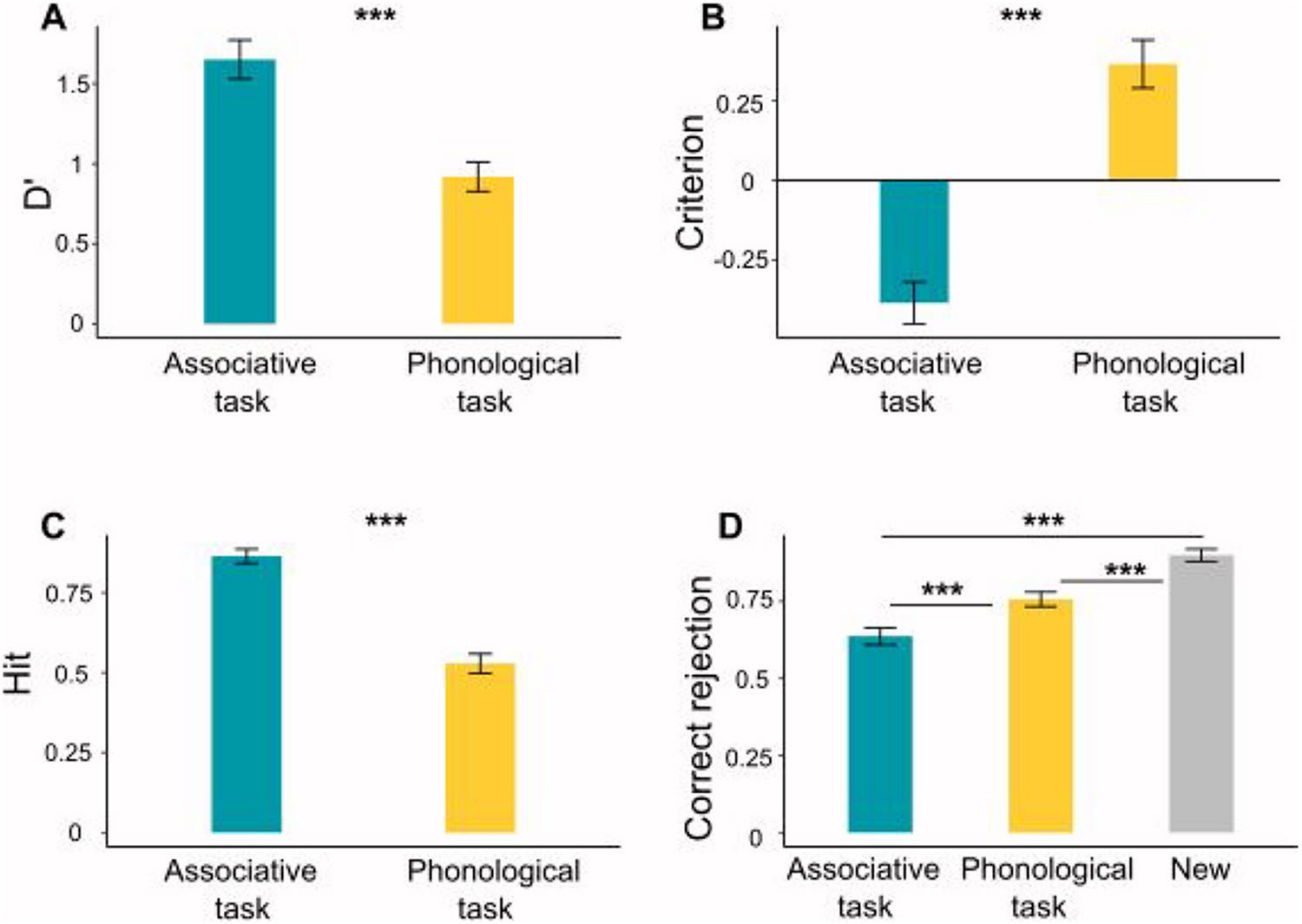
Scene memory performance in Experiment 1. **(A)** Sensitivity (D’); **(B)** criterion; **(C)** hit-rate; **(D)** correct rejection of the lures. The gray bar in D denotes correct rejection of completely new scenes. Error bars denote SEM; *** *p* < 0.001. Results show a consistent difference across memory measures between the associative thought task and the phonological fluency task

We tested our primary hypothesis by examining whether scene memory performance was influenced by the task that was performed following scene perception. Memory performance was first inspected by means of signal detection. Sensitivity (D’) and criterion were measured in each task condition by incorporating the scenes that appeared during the first stage, as well as the scenes that were gist related to the scenes that appeared at the first stage, separately for each cue condition (see *Methods*). A paired-samples *t*-test analysis revealed higher sensitivity for scenes that were followed by the associative thought task (D’ = 1.65, SD = 1.04) compared with scenes that were followed by the phonological fluency task (D’ = 0.91, SD = 0.79, *t*_(74,1)_ = 5.65, *p* < 0.001, d = 0.65; Fig. 2A). Additionally, scenes that were followed by the associative thought task had a significantly lower criterion (criterion = −0.38, SD = 0.56) compared with scenes that were followed by the phonological fluency task (criterion = 0.36, SD = 0.64, *t*_(74,1)_ = 9.35, *p* < 0.001, d = 1.1; Fig. 2B). Next, we separated memory of old scenes from correct rejection of new scenes. The hit rate for scenes that appeared prior to the associative thought task (mean = 0.86, SD = 0.19) was significantly higher than the hit rate for the scenes that appeared prior to the phonological fluency task (mean = 0.52, SD = 0.26, *t*_(74,1)_ = 12.85, *p* < 0.001, d = 1.4; Fig. 2c). Lastly, new scenes that appeared at test but did not share scene gist with scenes that appeared during the first stage and were excluded from signal detection and hit rate analyses, were used as baseline for estimating whether false alarm rate was influenced by task condition. A one-way ANOVA was conducted to compare false alarm rate for completely new images, and for scenes that were new but shared scene gist with images presented during the first stage of scene perception, separately for either of the tasks. This analysis revealed a significant main effect (F_(73,2)_ = 42.38, *p* < 0.001, partial eta square = 0.36; Fig. 2D), such that the false alarm rate was lowest for completely new scenes (mean = 0.1, SD = 0.07). This was followed by performance in gist-related scenes that preceded the phonological fluency task (mean = 0.25, SD=0.2), and the highest false alarm rate was observed for the scenes that preceded the associative thought task (mean = 0.37, SD = 0.24). Post hoc comparisons revealed that all conditions were significantly different from one another (associative thought vs. phonological fluency: *t*_(74,1)_ = 3.48, *p* < 0.001, d = 0.4; phonological fluency vs. new: *t*_(74,1)_ = 5.83, *p* < 0.001, d = 0.67; associative thought vs. new: *t*_(74,1)_ = 10.13, *p* < 0.001, d = 1.17; p-values are Bonferroni-corrected for multiple comparisons).

Together, these findings reveal that while scene memory hit rate is significantly improved following associative thought, one is nonetheless significantly more susceptible to making gist-related errors following associative thought.

In an aim to further probe the relationship between associative thought and scene memory, we examined whether the semantic distance between the chained-free associations provided for each scene predicts scene memory. To do that, we conducted a logistic mixed-model analysis, implementing trial-by-trial FF scores as a fixed variable and participants as a random variable, while attempting to predict memory hit rate performance. This analysis included only memory trials that depicted scenes that appeared following the associative thought probe at the first experimental stage. This modelling analysis did not yield significant results (estimate = 0.7, SE = 1.52, *t* = 0.46, *p* = 0.64, CI = [−2.28, 3.7]). We conducted an additional analysis to test whether trial-by-trial FF scores predict the false-alarm rate of new scenes that were gist related to previously viewed scenes. Here, too, trial-by-trial FF scores were used in the model as a fixed variable and participants as a random variable. This modelling analysis also did not yield significant results (estimate = −0.27, SE = 1.35, *t* =—0.2, *p* = 0.84, CI = [−2.29, 2.38]). Thus, we don’t find evidence that the enhancement effect of associative thought on scene gist-memory is dependent upon the magnitude of semantic distance involved during subsequent associative thought.

Lastly, we examined whether creativity is also associated with scene memory. Pearson correlation was computed between creativity scores and memory hit rate, specifically for scenes for which participants provided chained-free associations. This analysis did not yield significant results (*p* = 0.75). Similarly, computing the correlation between creativity and signal-detection parameters yielded null results (creativity-sensitivity: *p* = 0.1; creativity-criterion: *p* = 0.52). Thus, we do not find evidence that higher creativity is associated with better scene memory.

#### Semantic distance during associative thought correlates with creativity

**Fig. 3:**
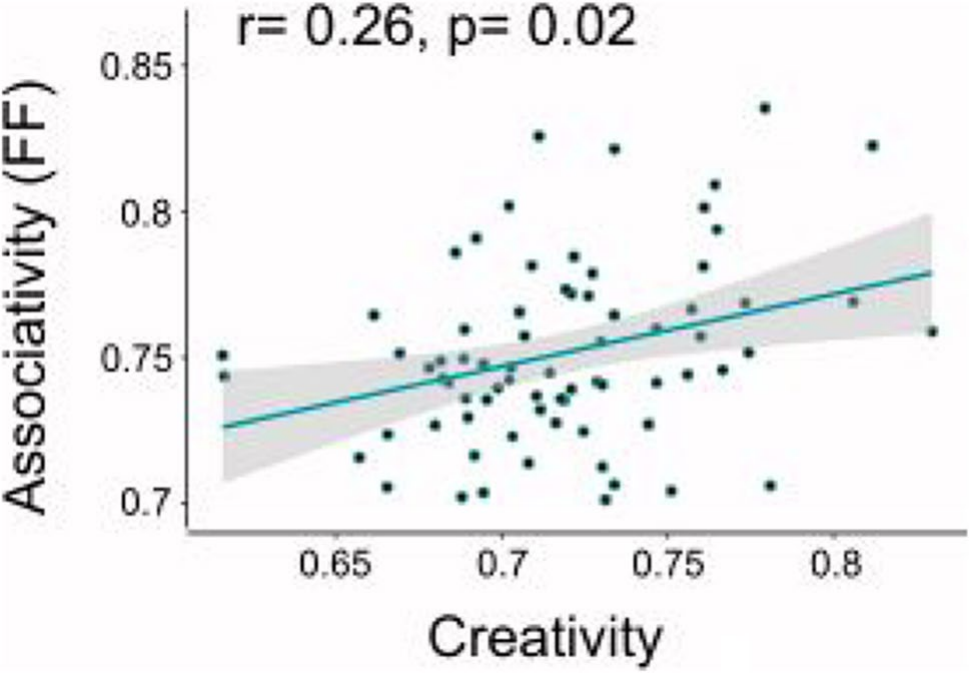
Semantic distance correlates with creativity in Experiment 1. Mean Forward Flow (FF) scores computed over participants’ chained-free associations correlate with creativity. Pearson *r* = 0.26, *p* = 0.02. Each dot denotes a participant

Our secondary endeavor was to examine whether associative thought following scene perception is linked with creativity. To test this, we correlated the FF score of associations provided during the associative thought probe with performance in the alternative uses task – which measured originality in divergent thinking as a proxy for creativity. This correlation analysis between the FF score in the associative thought task and the creativity score revealed that the greater the semantic distance in associative thought (i.e., the higher the FF), the higher the creativity score (*r* = 0.26, *p* = 0.02; Fig. 3).

The main results from Experiment 1 indicate that associative thought improves scene-gist memory. However, we cannot exclude the possibility that in the associative thought condition, participants retroactively labeled each scene before providing their chained-free associations, a process that is not implicitly required in the phonological fluency condition. To examine whether the effect of associative thought on scene gist memory is mediated by an implicit labeling process, we ran Experiment 2.

## Experiment 2

### General methods

Experiment 2 was very similar to Experiment 1 but required participants to first label the scene before they completed the associative thought or phonological fluency tasks. In this way, participants were explicitly required to begin each trial with scene labeling, regardless of the condition that followed. If indeed the effect of associative thought on scene gist memory that was found in Experiment 1 is elicited by an intermediate scene labeling process, the results in Experiment 2 should show a similar effect in both the associative thought and the phonological fluency conditions. Alternatively, if the effect found in Experiment 1 is independent from a potential labeling effect, then the results of Experiment 1 should be replicated in Experiment 2.

#### Participants

Ninety-eight participants took part in the experiment (mean age 40 years, SD = 14.25 years, 51 female). Eligibility criteria were identical to those of Experiment 1. Out of all participants, 74 participants successfully completed the experiment (mean age 41 years, SD = 14.5 years, 33 female). Participants who did not complete the experiment were excluded from the analyses. Similar to Experiment 1, Experiment 2 extended ~ 40 min and participants were paid 4 pounds for their time. The experiment was approved by the Institutional Review Board of Technion, Israel Institute of Technology.

#### Tasks and procedure

The tasks and procedure were identical to Experiment 1 with the only difference that following the presentation of each scene and after seeing the condition cue, participants were asked to first label the scene. For example, confined to a one-word label, participants often gave the label “canyon” to the scene depicting an overview of great, dry, mountains. Another example is the label “street” provided to an image depicting the typical view of tall buildings and yellow cabs in downtown Manhattan. Labels often capitalized on what the scene encapsulates (e.g., “playground,” “beach”), focused on the main object that appeared in the scene (e.g., “bridge,” “fireplace”), or depicted the activity typically performed in the scene (e.g., “bowling”). After explicit labeling, participants either provided four chained-free associations to that label in the associative thought condition or provided four words that begin with the same letter as the label they had given the scene in the phonological fluency condition.

#### Data analysis

Data analysis was identical to the analysis pipeline performed for Experiment 1.

### Results

First, we compared RT differences in the time it took to provide all five responses (a label and four additional responses) in the associative thought task and the phonological fluency task. A paired-samples *t*-test analysis revealed a null effect (associative thought task: mean RT = 39.76 s (SD = 50.89 s); phonological fluency task: mean RT = 40.21 s (SD = 31.08 s)), *t*_(73,1)_ = 0.98, *p* = 0.46, d = 0.01). This null effect replicates the findings from Experiment 1, and implies that the tasks are equivalent in their difficulty level.

**Fig. 4:**
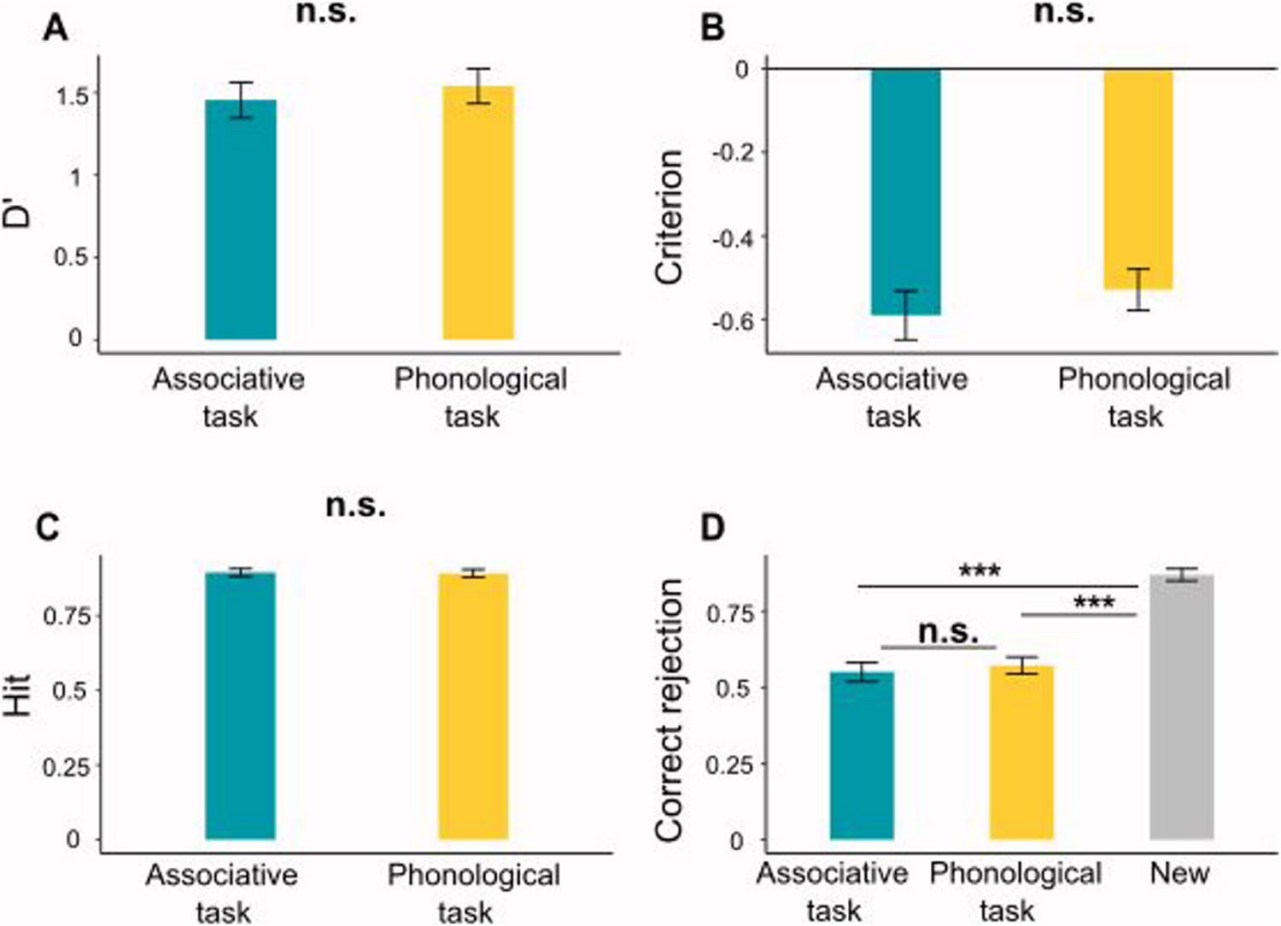
Scene memory performance in Experiment 2. **(A)** Sensitivity (D’); **(B)** criterion; **(C)** hit-rate; **(D)** correct rejection of the lures. The gray bar in D denotes correct rejection of completely new scenes. Error bars denote SEM; *** *p* < 0.001. Results do not replicate the difference found in Experiment 1 between memory performance in the associative thought task and the phonological fluency task

To test our primary hypothesis, we evaluated differences in memory performance between the associative thought and the phonological fluency tasks, closely following the analysis protocol of Experiment 1. A paired-samples *t*-test analysis revealed no difference between the conditions in their sensitivity (the associative thought task: D’ = 1.45, SD = 0.93; the phonological fluency task: D’ = 1.53, SD = 0.91; *t*_(73,1)_ = 0.74, *p* = 0.22, d = 0.08) (Fig. 4A). Additionally, scenes that were followed by the associative thought task did not have a significantly different criterion (criterion = −0.59, SD = 0.5) compared with scenes that were followed by the phonological fluency task (criterion = −0.52, SD = 0.42, *t*_(73,1)_ = 0.95, *p* = 0.17, d = 1.1; Fig. 4B). Next, we separated the memory of old scenes from correct rejection of new scenes. The hit rate for scenes that appeared prior to the associative thought task (mean = 0.89, SD = 0.11) were not significantly higher than the hit rate for the scenes that appeared prior to the phonological fluency task (mean = 0.89, SD = 0.11, *t*_(73,1)_ = 0.17, *p* = 0.43, d = 02; Fig. 4C). Lastly, a one-way ANOVA of false alarm rate revealed a significant main effect (F_(72,2)_ = 77.79, *p* < 0.001, partial eta square = 0.48; Fig. 4D), such that the false alarm rate was lowest for completely new scenes (mean = 0.13, SD = 0.17), followed by performance in gist-related scenes that preceded the phonological fluency task (mean = 0.42, SD = 0.23), and the highest false alarm rate was observed for the scenes that preceded the associative thought task (mean = 0.44, SD = 0.26). Post hoc comparisons, however, revealed that there was no significant difference between the associative thought task and the phonological fluency task in the rate of false alarm, *t*_(73,1)_ = 0.68, *p* = 0.25, d = 0.07. In both tasks, false alarm rate was significantly higher compared with completely new scenes (associative thought vs. new: *t*_(73,1)_ = 10.59, *p* < 0.001, d = 1.23; phonological fluency vs. new: *t*_(73,1)_ = 10.43, *p* < 0.001, d = 1.21; p-values are Bonferroni-corrected for multiple comparisons). In sum, when participants were asked to explicitly label the scenes, there were no longer significant differences between the tasks, and both tasks exhibited memory performance that is equivalent to the findings in the associative thought task in Experiment 1, with enhanced scene-gist memory and compromised memory for scene details.

Next, we examined whether the semantic distance between the chained-free associations provided for each scene predict scene memory. Similar to Experiment 1, we conducted a logistic mixed-model analysis, implementing trial-by-trial FF scores as a fixed variable and participants as a random variable, while attempting to predict memory hit rate performance, including only memory trials that depicted scenes that appeared following the associative thought probe at the first experimental stage. This modelling analysis did not yield significant results (estimate = 0.06, SE = 1.25, *t* = 0.05, *p* = 0.95, CI = [−2.4, 2.53]). We conducted an additional analysis that tests whether trial-by-trial FF scores predict false-alarm rate for scenes that are gist related to previously viewed scenes. Here, too, trial-by-trial FF scores were used in the model as a fixed variable and participants as a random variable. This modelling analysis also did not yield significant results (estimate = −1.16, SE = 1.2, *t* =—1.03, *p* = 0.3, CI = [−3.37, 1.04]). Lastly, conducting a correlation analysis between creativity and hit rate for scenes to which participants provided chained-free associations also yielded a null result (*p* = 0.17).

**Fig. 5:**
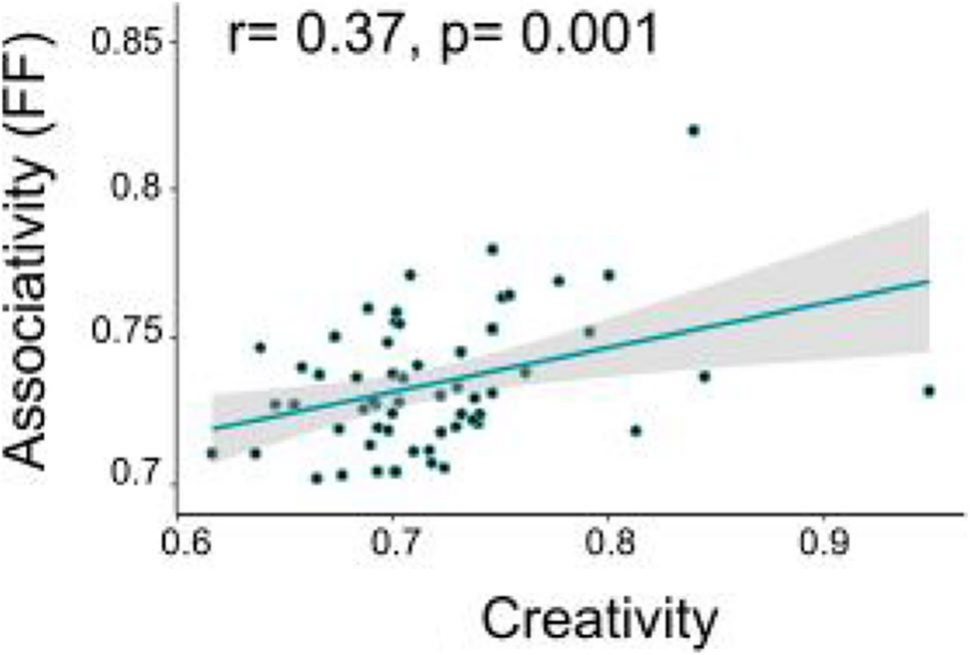
Semantic distance correlates with creativity in Experiment 2. Mean Forward Flow (FF) scores computed over participants’ responses in the phonological fluency task correlate with creativity. Pearson *r* = 0.37, *p* < 0.001. Each dot denotes a participant

Lastly, we found that similar to Experiment 1, the correlation between semantic distance during associative thought and creativity in Experiment 2 was significant as well. The greater the semantic distance in associative thought, the higher the creativity score (*r* = 0.37, *p* < 0.001; Fig. 5).

Taking both experiments together, the correlation between the breadth of associative thought and creativity is consistent across experiments and is not influenced by the implementation of the explicit-labeling requirement. The correlation between creativity and semantic distance of associative thought was found in the past when the FF score was computed for associations following word probes (Beaty et al., 2021; Gray et al., 2019; Merseal et al., 2023), and is replicated and extended here with relation to associations provided following scene image probes.

### General discussion

The primary hypothesis of Experiment 1 was that post-perceptual associative thought will facilitate scene memory, and indeed we find that in comparison to engaging in a control phonological task, associative thought facilitates scene memory sensitivity, as well as lowers one’s criterion. Although hit performance was higher following associative thought, gist-based false alarm rate was also higher, pointing to a specific enhancement in scene-gist memory, at the expense of scene details. In Experiment 2, we found that the effect of associative thought on scene gist memory was mediated by scene labeling. When participants labeled the scene prior to performing either the associative thought task or the phonological fluency task, scene gist memory was prioritized in both task conditions. The secondary hypothesis was that the semantic distance between associations during associative thought will be related to creativity, and indeed in both experiments we found a correlation between the FF score and creative performance in the alternative uses task. Finally, the hypothesis that high creativity will be linked with better scene memory was not supported by our findings in either of the experiments.

Associative thought, and the potential underlying implicit labeling, may facilitate gist memory in several ways. First, it may boost the encoding of contextual information at the expense of detail-related processing. This idea stems from the previous observations pointing to an overlap in neural activation between spontaneous associative thought and scene perception (Bar et al., 2007; Baror & He, 2021). This proposal also echoes a recent study (Melega & Sheldon, 2023) showing that when conceptual information is available during learning, even if implicitly, and learned items are associated to one another in their category, as is inherent in scene stimuli, subsequent memory is biased towards conceptual generalization at the expense of detail memory. The implicit “labelling” process believed to take place during associative thought may boost this process.

An alternative possibility is that associative thought, via labeling, simply prolongs the availability of gist information in mind. If this is the case, our findings complement past studies showing that gist-information is more readily accessed at scene onset (Torralba et al., 2006) by suggesting that in a similar manner, gist representation remains accessible for longer durations after scene offset, provided that one summarizes the scene-encoded information after that information is no longer available to the perceptual system. One related example is the phenomenon of “global precedence,” by which at image onset, the forest is more readily perceived compared with the trees (Hochstein & Ahissar, 2002; Kimchi, 1992; Miller & Navon, 2002; Navon & Norman, 1983). Scene labeling may potentially facilitate “global precedence” after scene-offset, and this process may take place implicitly during the natural emergence of spontaneous associative thought. Examining whether activity in scene-related brain regions is modulated during post-perceptual associative thought, and whether this processes is equivalent to neural modulations caused by scene labeling, would help in understanding the underlying mechanism of the effect associative thought has on scene memory, and whether implicit labeling does indeed take place.

A last alternative explanation of the results of Experiment 1 is that associative processing affects the levels of processing of the scene presented, regardless of the associative nature of the task. In other words, that associative thought boosts scene gist memory by deepening the levels of processing compared with phonological fluency. However, results from Experiment 2 reject such a levels of processing explanation because once producing the label, which we suggest elicited contextual associations of the scene, the results were equated between the conditions. The results of the phonological condition reflected the pattern seen with the associative task due to the labeling. A levels of processing explanation would have also predicted a correlation with semantic distance, i.e., the FF score, which we did not find.

Importantly, our findings bear relevance to the debate regarding the influence of spontaneous associative thought (i.e., mind wandering; Christoff et al., 2016; Mason et al., 2007; Seli et al., 2018), on memory. Mind wandering has often been characterized by disengagement from perceptual processing (Schooler et al., 2011), and therefore linked with reduced subsequent memory (Blonde et al., 2022). Nonetheless, recent works have shown that some forms of mind wandering are actually implicated with visual imagery and scene construction (Andrews-Hanna et al., 2013), which may aid scene representation when retrieval is needed. In another study, patients with damage to the hippocampus, a central DMN hub that is implicated in mind wandering, were less able to mentally represent visual images, shifting their mind wandering to be abstract rather than pictorial (McCormick et al., 2018). Healthy spontaneous associative thought seems to be intimately related to the ability to hold a scene in mind.

Thus, it is possible that spontaneous associative thought such as mind wandering in fact supports scene memory, as seen in our findings, when it fulfills two necessary conditions. First, that it is triggered after, rather than during, the perceptual experience, thus not compromising initial encoding. Second, that spontaneous thought is associated with, rather than unrelated to, the just experienced percept. In these conditions, mind wandering may facilitate globalized consolidation, and the prioritization of scene gist memory over memory of scene details of the just-perceived scene, by implicitly and retroactively creating gist-based mental “labels” or “tags,” which would be easily accessible later in time. This would be in line with previous suggestions, linking impaired spontaneous thought in elderly populations with impaired semantic scaffolding that is required for scene construction in memory (Mildner & Tamir, 2019), and would resolve the seeming contradiction between our findings, and findings reporting that mind wandering impairs memory (Blonde et al., 2022; Krimsky et al., 2017).

One promising avenue to further test this idea would be to examine eye movements during mind wandering following scene perception. Contrasting scene perceptual processing and mind wandering has recently been studied by means of eye tracking, aiming to use eye-related data to capture how scene perception is modulated when attention is shifted from processing the external input to attending internal spontaneous thought (Krasich et al., 2018, 2020, 2024; Zhang et al., 2021, 2022). These studies obtain eye-related indexes of mind wandering during scene viewing but can inspire future paradigms where saccade behavior during scene perception is compared to saccade behavior in subsequent rather than parallel spontaneous thought. This has the potential to elucidate whether saccade-related behavior after scene perception reflects revisiting the just-observed scene, and whether such behaviour also relates to subsequent memory.

Another point of discussion should address the relation between our current findings and past studies that proposed that associative thought and scene perception converge on associative processing. In a study by Baror et al. (2022), it was shown that upholding an associative mindset promotes processing of associations embedded within parallel-presented scenes. Associative thought was suggested to sustain an associative processing mode, which is compatible with the associative processes involved during scene perception, boosting overall associative and contextual processing. The fact that scene memory was here facilitated by associative thought but was not predicted by trial-by-trial variability in semantic distance encapsulated in the provided associations lends support to this global “mindset” idea. That said, our findings in the current study suggest that the connection between associative thoughts and scene memory is mediated by implicit scene labelling. It is not unlikely that a similar process of implicit “labelling” was facilitated when engaging in an associative mindset. This possibility was not raised in Baror et al. (2022). That said, it is worth noting that some fundamental differences exist between the two studies, which may render this explanation too simplistic. First, Baror et al. (2022) manipulated an associative mindset in a fixed manner across participants rather than allowing them to form their individual, spontaneous associations as was done here. As opposed to the current experiment, these fixed associations were not related to the presented scenes. Second, the task was focused on identifying differences between a current and a previously presented scene, rather than testing scene memory as was done here. It is still possible that an associative mindset facilitates scene perception via a shared associative mechanism, and that later scene memory is facilitated by spontaneous associative thought through the implicit generation of scene labels. Future studies should directly test the link between the two phenomena and examine whether scene labelling mediates the previously found link between an associative mindset and improved scene perception.

Our secondary finding shows that creativity correlated with semantic distance as measured by FF scores in the associative thought task is in line with the account that couches creativity as part of a broader set of spontaneous-thought phenomena (Christoff et al., 2016; Girn et al., 2020). Indeed, it has been shown in the past that creativity is tightly linked with the distance one travels over their semantic memory during associative tasks (Beaty & Kenett, 2023; Beaty et al., 2021; Benedek et al., 2023; Gray et al., 2019; Kenett, 2023, 2024). Past demonstrations used linguistic stimuli as probes for the free-associations process (Beaty et al., 2021; Gray et al., 2019). Our findings replicate and extend this work to scene-based associations.

### Limitations

Finally, we acknowledge that there are a few limitations to our study. First, while we used predetermined scene labels in Experiment 1, as they are provided by the “SuN” database, to compute the FF score, it is nonetheless possible that participants generated their own individual labels during the experiment, which might have ultimately influenced their FF scores and their found relationship to creativity. This, however, is not likely to interfere with the FF score, as it showed the same correlation with creativity in Experiment 2 where participants generated their own scene labels. Second, although we tried to simulate spontaneous associative thought, participants in the task were nonetheless required to provide exactly five words in the chained-free associations task, which limited participant’s “spontaneity.” This requirement was aimed at standardizing the number of associations provided but may have downscaled the variability in associative thought within and across participants, potentially influencing the semantic distance involved between associations. Future research is needed to generalize our results in contexts where associative thought is completely spontaneous, while utilizing the necessary measures to meet the intra- and inter-subject variability that would be inevitably involved.

### Conclusions

In sum, this study reveals that associative thought following scene perception likely enhances scene-gist memory through an implicit scene-labeling process. The study also shows that the semantic distance elicited during scene-based associative thought correlates with creative abilities. Together, these findings point to a cross-modal role of spontaneous associative thought in memory and cognition, and call for further uncovering the beneficial functions spontaneous associative processes may serve in everyday perception and memory experiences.

## Data Availability

Raw anonymized data and experiment materials can be found in via the Open Science Framework at: https://osf.io/uwqyv/?view_only=b94ab4ca2cad408899e660c04188c70d

## Code availability

Code is openly available at: https://osf.io/uwqyv/?view_only=b94ab4ca2cad408899e660c04188c70d
